# [Retracted] miR-222 regulates brain injury and inflammation following intracerebral hemorrhage by targeting ITGB8

**DOI:** 10.3892/mmr.2026.13841

**Published:** 2026-03-09

**Authors:** Yan-Yan Bai, Jun-Zhi Niu

Mol Med Rep 21: 1145–1153, 2020; DOI: 10.3892/mmr.2019.10903

Following the publication of this paper, it was drawn to the Editor's attention by a concerned reader that most of the flow cytometric (FCM) assay data featured in Figs. 2C and 6C were strikingly similar to FCM data which were ultimately published in a number of other papers in different journals that were written by different authors at different research institutes, including a paper that was submitted on an earlier date to the same journal (*Molecular Medicine Reports*).

Owing to the fact that the contentious data in the above article were found to be strikingly similar to data that have appeared elsewhere in other papers in the scientific literature, the Editor of *Molecular Medicine Reports* has decided that this paper should be retracted from the Journal. The authors were asked for an explanation to account for these concerns, but the Editorial Office did not receive a reply. The Editor apologizes to the readership for any inconvenience caused.

